# How geometry shapes division of labor

**DOI:** 10.7554/eLife.63328

**Published:** 2020-11-03

**Authors:** Merlijn Staps, Corina Tarnita

**Affiliations:** Department of Ecology and Evolutionary Biology, Princeton UniversityPrincetonUnited States

**Keywords:** reproductive specialization, evolution, topology, None

## Abstract

A mathematical model shows how the shape of early multicellular organisms may have helped cells evolve specialized roles.

**Related research article** Yanni D, Jacobeen S, Márquez-Zacarías P, Weitz JS, Ratcliff WC, Yunker PJ. 2020. Topological constraints in early multicellularity favor reproductive division of labor. *eLife* **9**:e54348. doi: 10.7554/eLife.54348

Our body is built from cells with dedicated roles: red blood cells transport oxygen, retinal cells detect light, and immune cells fight off pathogens. However, the earliest multicellular organisms did not have such specialized cells, and how the division of labor between cells first evolved remains unknown (Bonner, 2000; Brunet and King, 2017; van Gestel and Tarnita, 2017).

One of the best-studied examples of division of labor is between germ cells, which reproduce, and somatic cells, whose sole purpose is to ensure that the germ cells survive. Differentiation between germ and somatic cells has evolved repeatedly, and occurs even in simple multicellular organisms with far fewer cell types than animals, such as green algae or social amoebae. Because it is impossible to determine what selective pressures drove the evolution of germ-soma differentiation hundreds of millions of years ago, biologists have turned to mathematical models to understand how germ and soma cells came about (Gavrilets, 2010; Michod, 2007).

Models for the evolution of germ-soma differentiation start from the assumption that cells within a multicellular group can invest resources into the group’s survival, reproduction, or a combination of both. Using these models, researchers can ask what conditions allow specialized cells that only invest in reproduction (germ) or survival (soma) to evolve. Previous work revealed that division of labor can only evolve under stringent conditions where specialized cells have to be better (i.e. more efficient) at their job than non-specialized cells (Michod, 2007). But, these conditions may not necessarily have been met early on in the evolution of division of labor.

Now, in eLife, Peter Yunker, William Ratcliff and colleagues at the Georgia Institute of Technology – including David Yanni and Shane Jacobeen as joint first authors, Pedro Márquez-Zacarías and Joshua Weitz – report that the geometry of certain early multicellular organisms may have made it easier for division of labor to evolve (Yanni et al., 2020). The team developed a model for germ-soma differentiation that incorporates spatial structure. While earlier models assume survival investments are pooled together and shared amongst all cells, the model created by Yanni et al. assumes that a cell’s investment in survival is only shared with immediate neighbors (Figure 1). In this setup, the shape of the multicellular group plays a crucial role as it dictates which cells are neighbors.

**Figure 1.**
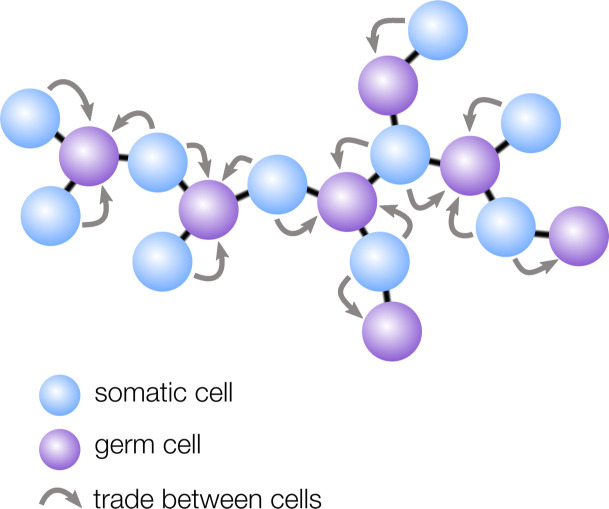
How geometry influences cell differentiation. Many multicellular organisms have evolved germ-soma differentiation — a division of labor between germ cells, specialized for reproduction, and somatic cells, which help the organism survive. Yanni et al. show that multicellular organisms with a sparse cellular geometry, such as the structure shown here, are more likely to evolve germ-soma differentiation. In such organisms, germ cells (purple) can alternate positions with somatic cells (blue), so the survival investments made by somatic cells exclusively benefit germ cells (gray arrows).

Yanni et al. found that ‘sparse’ geometries in which cells have few neighbors — such as filaments and trees — are particularly conducive to the evolution of germ-soma differentiation. In these structures, regularly spaced cells take on the role of germ, while the interspaced cells become somatic to support the reproductive cells (Figure 1). The survival investments made by somatic cells are therefore now exclusively shared with germ cells, rather than with all cells in the group, including with other somatic cells. Yanni et al. showed that this efficient sharing of survival benefits relaxes the conditions under which division of labor can evolve: in sparse multicellular geometries, division of labor can even be favored when specialized cells are slightly less efficient than non-specialized ones.

Intriguingly, in many existing multicellular organisms, the spatial organization of germ and somatic cells mimics the pattern predicted by the model. For example, in cyanobacteria the role of somatic cells is taken on by specialized nitrogen fixers that are regularly spaced along filaments to support the surrounding reproductive cells (Flores and Herrero, 2010). And while complex multicellular organisms — which are beyond the reach of this model — typically do not have regularly spaced germ cells, glimpses of the predicted organization can still be seen. For instance, fruit fly egg cells develop from a cluster of interconnected cells of which only one becomes the egg, while the surrounding cells adopt a supporting role (Bastock and St Johnston, 2008; Alsous et al., 2018).

While models such as the one by Yanni et al. shed light on the evolutionary forces that shape cell differentiation, they tell us little about the underlying mechanisms (Márquez-Zacarías et al., 2020). These findings, however, provide a promising lead: if germ-soma differentiation is associated with a specific spatial organization, then its evolution requires developmental mechanisms that allow cells to differentiate according to their location. A future goal is then to understand how such developmental mechanisms originated in evolution.
